# Phenotypic plasticity increases exposure to extreme climatic events that reduce individual fitness

**DOI:** 10.1111/gcb.16663

**Published:** 2023-03-19

**Authors:** Charlotte E. Regan, Ben C. Sheldon

**Affiliations:** ^1^ Department of Biology Edward Grey Institute, University of Oxford Oxford UK

**Keywords:** extreme climatic event, great tit, laying date, rainfall, reproductive success, temperature, Wytham woods

## Abstract

Climate models, and empirical observations, suggest that anthropogenic climate change is leading to changes in the occurrence and severity of extreme climatic events (ECEs). Effects of changes in mean climate on phenology, movement, and demography in animal and plant populations are well documented. In contrast, work exploring the impacts of ECEs on natural populations is less common, at least partially due to the challenges of obtaining sufficient data to study such rare events. Here, we assess the effect of changes in ECE patterns in a long‐term study of great tits, near Oxford, over a 56‐year period between 1965 and 2020. We document marked changes in the frequency of temperature ECEs, with cold ECEs being twice as frequent in the 1960s than at present, and hot ECEs being ~three times more frequent between 2010 and 2020 than in the 1960s. While the effect of single ECEs was generally quite small, we show that increased exposure to ECEs often reduces reproductive output, and that in some cases the effect of different types of ECE is synergistic. We further show that long‐term temporal changes in phenology, resulting from phenotypic plasticity, lead to an elevated risk of exposure to low temperature ECEs early in reproduction, and hence suggest that changes in ECE exposure may act as a cost of plasticity. Overall, our analyses reveal a complex set of risks of exposure and effects as ECE patterns change and highlight the importance of considering responses to changes in both mean climate and extreme events. Patterns in exposure and effects of ECEs on natural populations remain underexplored and continued work will be vital to establish the impacts of ECEs on populations in a changing climate.

## Graphical abstract

Understanding the potential impacts of climate change on wild animal populations requires knowledge on the effects of changes in mean climate and extreme climatic events (ECEs), though the latter have received less attention to date. Here, we show that increased exposure to high and low temperature ECEs often reduces great tit reproductive, and that birds now encounter more ECEs than they did in the past. Increased exposure to extreme low temperature events is partly driven by advances in laying date and the negative impacts of such extreme cold events on reproductive success may act as a cost of plasticity.

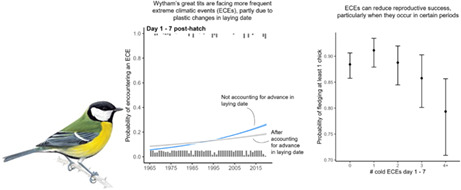

## 1 INTRODUCTION

Climate change is widely recognized as a major threat to biodiversity and a wealth of research has demonstrated the impacts that changes in average climate conditions can have on population dynamics (Post et al., 2009), species distributions (Parmesan & Yohe, 2003), and community structure (Walther et al., 2002). However, climate change is also expected to alter climate variability, including the frequency, duration, and severity of extreme climatic events (ECEs) (Seneviratne et al., 2012). Indeed, we are already seeing pronounced changes in the patterns of ECEs across the globe, such as increases in the frequency and duration of heatwaves (Perkins‐Kirkpatrick & Lewis, 2020) and in the frequency and severity of extreme precipitation events (Myhre et al., 2019).

The responses of populations and species to climate change are likely to be determined both by the independent effects of changes in the mean climate and in the patterns of ECEs, as well as their interaction (Lawson et al., 2015). Recent work has even suggested that changes in climate variability may have disproportionately larger effects on species than changes in mean conditions (Vasseur et al., 2014), potentially because increased variability results in individuals experiencing a range of temperatures of the same magnitude as changes in average temperature over many decades, with such temperatures being more likely to exceed a species' physiological limits (Lawson et al., 2015). Consequently, establishing how populations respond to variation in climate extremes will be crucial for understanding the ecological impact of current and future climate change, thereby informing actions designed to mitigate against climate change impacts.

Despite growing awareness of the threats that changes in the patterns of ECEs pose to biological systems, we still have a limited understanding of the ways in which ECEs impact natural populations. In recent years, several studies have highlighted the potential for ECEs to have marked consequences for survival (Gardner et al., 2017; Tanner et al., 2017) and reproductive success (Marcelino et al., 2020) and that such effects can affect selection on traits such as reproductive timing (Marrot et al., 2017) and migratory behaviour (Acker et al., 2021). Although we are beginning to gain insights on what ECEs mean for natural populations, there are substantial gaps in our knowledge, such as the time dependence of ECE effects on fitness (Bailey & van de Pol, 2016; Smith, 2011), the potential interactions between different types of ECEs (Smith, 2011), and the role of ECE frequency over and above the presence/absence of single events (Bailey & van de Pol, 2016; Smith, 2011).

Studying the biological consequences of ECEs is challenging, with this likely being behind the relative lack of work investigating the consequences of ECEs compared to literature examining the effects of changing average climate. ECEs are commonly classified according to whether a weather/climate variable exceeds a threshold value at the upper or lower end of observed values of the variable in question (Seneviratne et al., 2012). Thus, they are largely defined by their extremity. As a result, ECEs tend to be rare over the timescales that we frequently study populations. The rarity of ECEs poses significant challenges in terms of ruling out the effects of confounding variables (Altwegg et al., 2017) and gaining sufficient statistical power to reliably quantify their impacts both in the short and long term. This is not to say that insights cannot be gained from one‐off events (e.g. Grant & Grant, 1993; see Altwegg et al., 2017 for a discussion of the value of single events) but depending on their severity, investigations into the consequences of ECEs may be hampered by the availability of the necessary data. Similarly, understanding how changes in the patterns of ECEs with climate change will impact populations in the future requires capturing variability in the occurrence of ECEs—something that clearly necessitates capturing multiple events.

Due to the challenges associated with studying ECEs in natural populations, study systems where there are both long‐term fitness and climatic data offer a valuable opportunity to begin to fill some of the gaps in our knowledge of how changing patterns of ECEs under climate change might affect population health and persistence, and evolutionary change. The study of great tits (*Parus major*) in Wytham Woods, Oxfordshire, UK, is one such study for which the available data put such questions within reach. With standardized data spanning more than 50 years and more than 19,000 breeding attempts, it is possible to both explore changes in the exposure of birds to ECEs and ask questions about the effects of ECEs on reproductive success. Furthermore, great tit populations, including the one in Wytham Woods, have been the focus of considerable research aimed at understanding how changing average climate conditions affect reproductive behaviour and performance (Ahola et al., 2009; Bordjan & Tome, 2014; Bryan & Bryant, 1999; Simmonds et al., 2017). This prior work provides an ideal foundation on which to examine the effects of other climatic changes, such as changes in climatic variability.

In this study, we had two overarching aims. First, we aimed to characterize temporal patterns in temperature and rainfall ECEs coinciding with reproduction, both in terms of their raw frequencies and in terms of the likelihood of individual exposure to ECEs given that mean laying date in this population has advanced over time (Charmantier et al., 2008; Cole et al., 2021). Second, we aimed to understand the impact of extreme temperature and rainfall events on great tit reproductive success, with a particular focus on understanding whether the impacts of ECEs were dependent on the breeding stage at which they occurred, whether there was evidence for cumulative effects of ECEs, and whether different types of ECEs had interactive effects on reproductive success.

## 2 METHODS

### 2.1 Study population

The great tit population in Wytham Woods, near Oxford, UK, has been intensively studied using standardized methods since 1947 (Perrins & McCleery, 1989) and since 1961, birds breeding in 996 nestboxes at fixed locations have been monitored (an additional 21 boxes were added in 1977). Nestboxes are visited at least once per week during the breeding season (from early April to June), enabling the collection of detailed data on breeding attempts, including laying date (the date when the first egg is laid), clutch size, hatching date and hatching success, and fledging success for each pair. We chose to define the beginning of the study period for this paper as 1965 to allow for equilibration of the population to the provision of new nest boxes which was completed in 1961 (mean generation time of great tits is <2 years: Bouwhuis et al., 2009).

### 2.2 Defining ECEs

We opted to explore the effects of both the occurrence and frequency of three types of ECE: (i) extreme low temperature events; (ii) extreme high temperature events, and (iii) extreme high rainfall events. In each case, an ECE was defined as an event with an empirically observed occurrence in the extreme 5% of the tail of the relevant distribution over the study period (1965–2020) (Marrot et al., 2017). To establish these cut‐offs, we calculated the deviations between daily mean temperature/rainfall and average temperature/rainfall in the relevant calendar month between 1965 and 2020 (using different time windows produced comparable results—see Supporting information). Note that, in doing so, we assume implicitly that deviations of the same magnitude at different points over the course of the study are potentially equivalent in their effects. We obtained temperature and rainfall data from Met Office Hadley Centre datasets (https://www.metoffice.gov.uk/hadobs/). In the case of temperature, we used the Central England Temperature dataset, whilst for rainfall we used data interpolated onto a 5 km × 5 km grid and extracted the data corresponding to the grid square containing Wytham. We used the 5th percentile of the temperature distribution to define a cold ECE (≤ − 4.45°C below the monthly mean temperature) and the 95th percentile to define a hot ECE (≥ + 4.51°C above the monthly mean temperature). For rainfall, we used the 95th percentile to define a high rainfall ECE (≥7.32 mm rain in 24 h). Hence, an ECE was defined as having occurred on each day that these extremes were observed. Therefore, according to our definition, an extreme event lasting multiple days would be counted as multiple ECEs. We opted to use a climatic definition instead of a biological definition as our interest in the time dependence of ECE effects on reproductive success meant we required a definition that could be used across time periods and that ensured events were frequent enough to enable analysis. However, we note that approaches for defining ECEs are still debated (Bailey & van de Pol, 2016; Smith, 2011).

To understand whether the timing of ECEs was important in mediating their effect on reproduction in this system, we split each breeding attempt into four time periods. The first (hereafter Period 1) covered the days between the start of incubation (assumed to start on the day females laid their last egg) and the day the eggs hatched. Then, as in (Marrot et al., 2017), we split the period post‐hatching into Period 2 (hatch day to Day 7 post‐hatch to capture the period when chicks are unable to thermoregulate), Period 3 (Days 8–Day 15 post‐hatch when chick food requirements are at their highest), and Period 4 (Days 16–21 with fledging occurring on or shortly after Day 21). Thus, for each breeding pair between 1965 and 2020, we calculated the presence/absence and the frequency of each type of ECE in each of the four periods. These periods are therefore of different durations, with Period 1 being variable (depending on incubation speed, with incubation duration having increased by 1 day over the course of study). However, our focus is on impacts on specific developmental stages, not the direct comparison of frequency of events between stages.

### 2.3 Statistical analysis

All analyses were conducted in R version 4.0.3 using the package ‘brms’ (Bürkner, 2017, 2018).

#### 2.3.1 Temporal trends

We first asked how the frequency of each type of ECE changed over the course of the study. In this case, we calculated the number of ECEs (defined as above) occurring between 1 April and 30 June of each year between 1965 and 2020 (>99.9% of clutches were started on or after 1 April). We selected these months as they cover >99% of first clutches within the Wytham population. We then used these counts as the response variable in a GLM with year as the single covariate, assuming negative binomial errors.

Given that we know mean laying date in this population has advanced over time (Charmantier et al., 2008; Cole et al., 2021), we also examined temporal patterns in the exposure of birds to each type of ECE. First, we looked at how the probability of a bird experiencing at least one of each type of ECE in each of the four breeding periods had changed between 1965 and 2020. To do this, we ran a model for each type of ECE in each of the four periods, with presence/absence of the ECE as the response variable and year as the single covariate, with Bernoulli error distribution. Second, we wanted to understand whether any temporal changes in the exposure of birds could be explained by temporal changes in laying date—that is, whether an advancement of breeding time might lead to an elevated risk of experiencing an ECE. Thus, we re‐ran the above analysis, but this time included an individual's January laying date (i.e. 1 = 1 January) as an additional fixed effect.

#### 2.3.2 Fitness effects of ECEs

To examine the reproductive consequences of ECEs, we used different metrics for pre‐hatching and post‐hatching periods. When looking at the effects of ECEs occurring during Period 1 (incubation to hatching), we looked at the consequences for the proportion of a clutch that hatched (hereafter hatching success). For the three periods post‐hatching, we looked at both the probability that birds fledged at least one chick (hereafter brood success) and the proportion of hatched chicks that a pair fledged (hereafter fledging success). We included only first clutches in our analysis by excluding breeding attempts where the laying date was >30 days after the first clutch of the year (as in van Noordwijk et al., 1995). Similarly, we removed all clutches that had been subject to experimental manipulation due to the possible effects of such manipulations on reproductive success.

Models included the measure of reproductive success as the response and both year‐centred laying date and either the presence/absence or number of ECEs as fixed effects (note that we only present results using the number of ECEs in the main text). Each type of ECE was considered separately due to collinearity between low and high temperature ECE frequencies. For ECE frequency, we opted to collapse higher frequencies into one value and subsequently treat frequency as a categorical variable rather than as a covariate (see Table S1 for grouping information). We chose to group higher ECE frequencies in this way (i) due to the extreme rarity of breeding attempts encountering very high ECE frequencies and thus difficulties in estimating relationships between fitness and these values. By combining the highest frequencies into one category, we were able to enhance the power to detect an effect of ECE frequencies above a certain value. (ii) because treating ECE frequency as a categorical variable made it more straightforward to assess whether there was a given frequency above which impacts on fitness were more pronounced. Models for both hatching success and fledging success assumed a binomial distribution, whilst models of brood success assumed a Bernoulli distribution. In both cases, we included nestbox identity and breeding year as random effects. For all models, we used weakly informative priors for fixed effects (normal [0,1]) and random effects (cauchy [0,5]), with visual prior predictive checks used to ensure that all biologically plausible outcomes (e.g. hatching success ranging from 0 to 1) were possible under our priors.

Models of both hatching success and fledging success were run for 10,000 iterations across four chains with a warm‐up of 2000 iterations and a thin of 10 iterations. Models of brood success were run for 2000 iterations across four chains, with a warm‐up of 500 iterations and a sample taken each iteration. In each case, we selected these values to ensure convergence and good effective sample sizes.

#### 2.3.3 Interactions between ECE types

We also wanted to understand whether the effects of hot and cold ECEs varied if there was also a high rainfall ECE in the same period. Given the rarity of each type of ECE, we were only able to consider how the effects of cold and hot ECEs depended on the presence/absence of high rainfall ECEs. Thus, we examined interactions between the presence/absence of hot and cold ECEs and the presence/absence of rainfall ECEs in each period, as well as interactions between the frequency of hot and cold ECEs and the presence/absence of high rainfall ECEs. Models had the same structure as above but included interactions in the place of main effects.

For each analysis, we assessed model convergence using trace plots, by assessing effective sample sizes, and using the Gelman–Rubin convergence diagnostic (Rhat = 1). We defined effects as statistically significant when the 95% credible intervals did not overlap zero.

## 3 RESULTS

### 3.1 Temporal patterns

**FIGURE 1:**
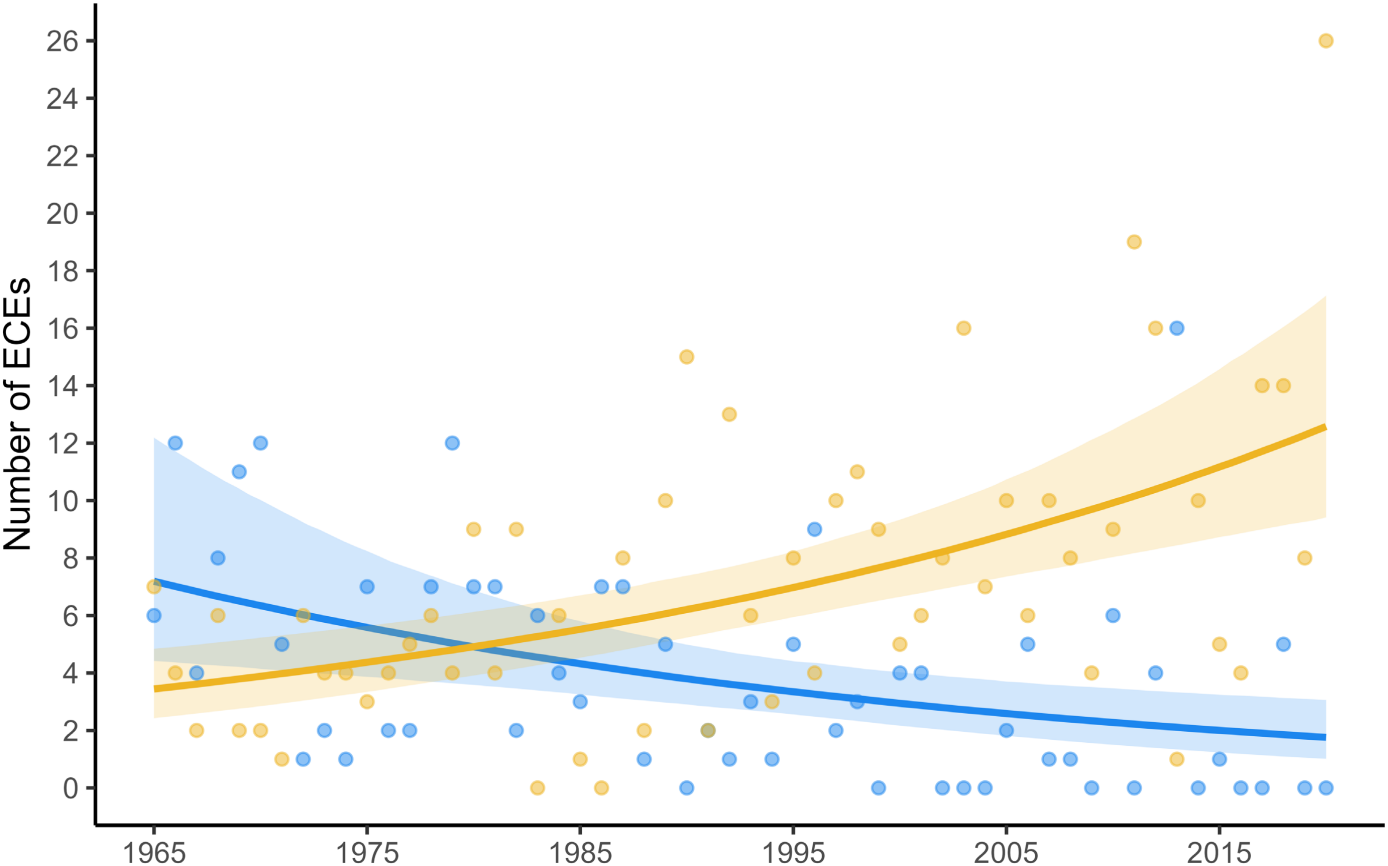
Fitted values for change in the number of hot (yellow) and cold (blue) extreme climatic events experienced by great tits between 1 April and 30 June, for Wytham, near Oxford UK, between 1965 and 2020 (*N* = 56 years). Whilst the number of cold extreme climatic events (ECEs) over the breeding season (April–June) has decreased over time, the number of hot ECEs in the same period has increased.

**FIGURE 2:**
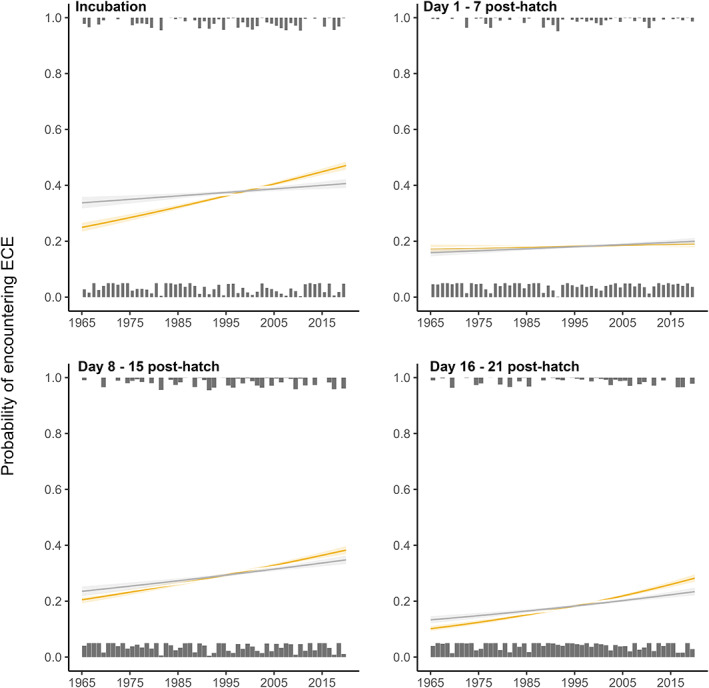
Shown are temporal trends in the probability of exposure of great tits to hot extreme climatic events (ECEs) across different periods of the breeding cycle (*N* = 12,219 breeding attempts). Grey bars correspond to the proportion of birds in each year that did (top) or did not (bottom) encounter a hot ECE. Coloured lines represent temporal trends before laying date was accounted for in the model, whilst grey lines correspond to model predictions when individual laying dates were included as fixed effect.

**FIGURE 3:**
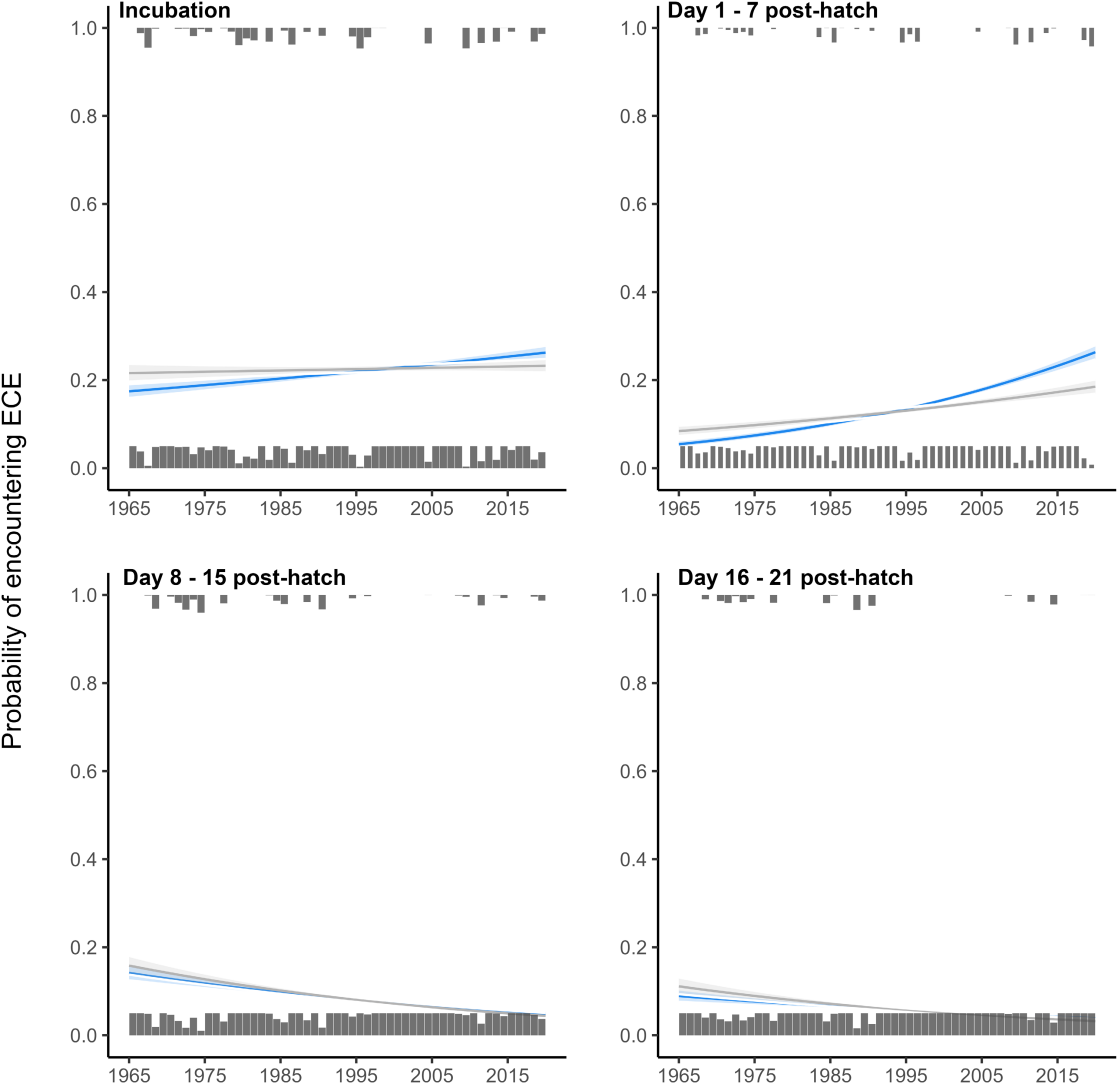
Shown are temporal trends in the probability of exposure of great tits to cold extreme climatic events (ECEs) across different periods of the breeding cycle (*N* = 12,219 breeding attempts). Grey bars correspond to the proportion of birds in each year that did (top) or did not (bottom) encounter a cold ECE. Coloured lines represent temporal trends before laying date was accounted for in the model, whilst grey lines correspond to model predictions when individual laying dates were included as fixed effect.

There have been marked temporal changes in the frequencies of hot and cold ECEs between 1965 and present. Hot ECEs were, on average, ~three times more frequent between 2010 and 2020 than between 1965 and 1975 (Figure 1). In contrast, cold ECEs were, on average, ~twice as frequent between 1965 and 1975 than they were from 2010 onwards (Figure 1). These trends were largely explained by the temporal change in mean temperature, with the slopes of both trends being reduced once mean spring temperature was accounted for (from est = −0.02 [95% CI = −0.04 – 0.00] to −0.00 [−0.02–0.01] for cold ECEs, and from est = 0.02 [95% CI = 0.01–0.04] to 0.01 [−0.00–0.02] for hot ECEs). In line with the increase in hot ECE frequency noted above, we found that birds are now more likely to experience a hot ECE in all intervals between incubation and fledging, except between hatching and chicks being 7 days old (Figure 2). However, in contrast to the overall decrease in the frequency of cold ECEs over time, we found that birds breeding in recent years are in fact more likely to encounter cold ECEs in the early stages of breeding (i.e. between incubation and hatching and between hatching and chicks reaching 7 days of age; Figure 3). Accounting for laying date generally reduced the slopes of temporal trends, particularly during the incubation period and the first 7 days post‐hatch (Figures 2 and 3). Overall, this suggests that the changes in exposure to hot and cold ECEs over time have been at least partly due to temporal changes in laying date behaviour.

### 3.2 Fitness effects of ECEs

#### 3.2.1 Hatching success

**FIGURE 4:**
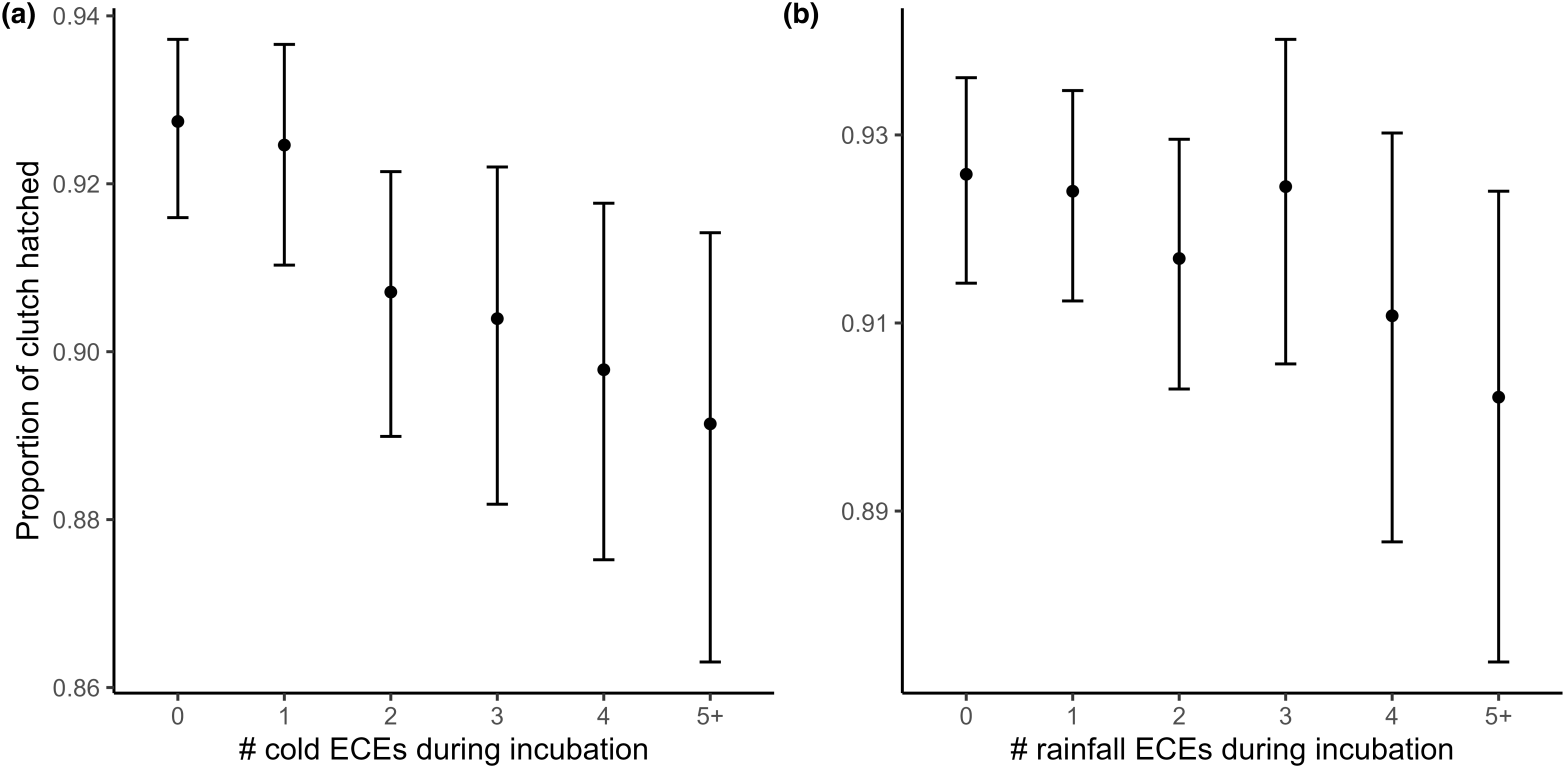
The proportion of great tit eggs that hatched declined as (a) the number of cold extreme climatic events (ECEs) and (b) the number of high rainfall ECEs increased (*N* = 11,339 breeding attempts). Shown are model predictions and associated 95% credible intervals.

The proportion of eggs that pairs hatched was negatively impacted by cold ECEs whilst there was no evidence for any effect of experiencing a hot ECE or a high rainfall ECE, with all credible intervals spanning zero (Table S2). Effects of both cold and high rainfall ECEs were more pronounced when accounting for the frequency with which individuals encountered such events, with hatching success decreasing by 4 percentage points (corresponding to a 55% increase in the hatching failure rate) when comparing individuals that did not experience a cold ECE to those that experienced 5+ cold ECEs (Figure 4a). Similarly, hatching success declined by 2.4 percentage points (corresponding to a 51% increase in hatching failure rate) when comparing individuals that encountered no rainfall ECEs to those that encountered five or more (Figure 4b).

#### 3.2.2 Brood failure

**FIGURE 5:**
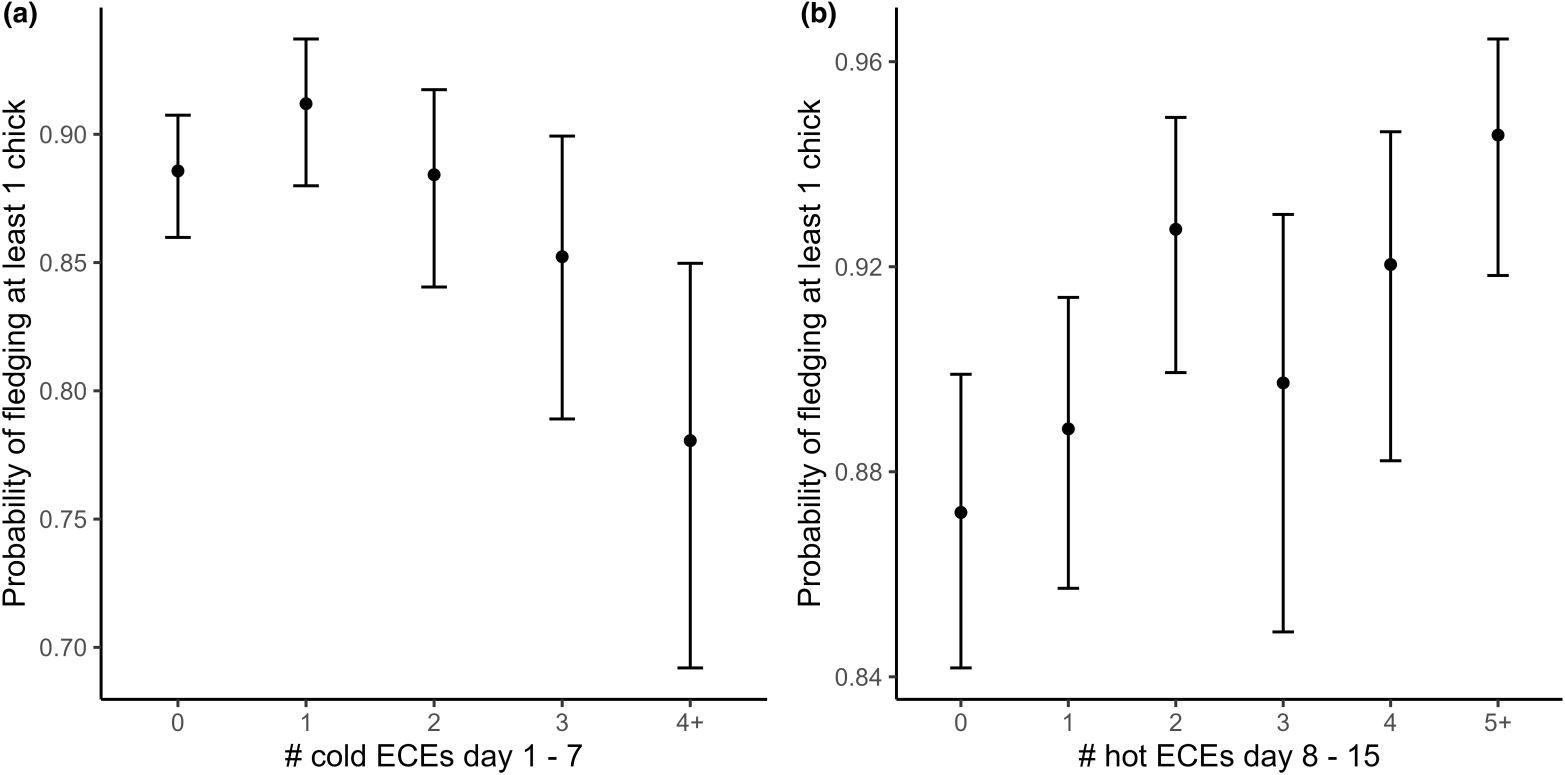
(a) The probability of great tit pairs fledging at least one chick declined as the number of cold extreme climatic events (ECEs) experienced from hatching to Day 7 post‐hatch increased. (b) In contrast, pairs were more likely to fledged at least one chick if they experienced multiple hot ECEs during the period of peak food requirement (Days 8–15 post‐hatch) (*N* = 11,264 breeding attempts). Shown are model predictions and associated credible intervals.

When examining the probability of pairs fledging at least one chick, we found evidence for effects of both cold and hot ECEs, though not in all the three post‐hatch time periods (Table S3), and effects varied in direction. Furthermore, in contrast to hatching success, there was no evidence for an effect of high rainfall ECEs, regardless of the period we considered (Table S3). We found that the likelihood of pairs fledging at least one chick declined from 88.6% (95% CI: 86.0–90.7) for birds that did not encounter a cold ECE between Days 1 and 7 post‐hatch to 78.1% (95% CI: 69.2–85.0) for those birds that encountered four or more cold ECEs within this 7‐day period (Figure 5a). Similarly, the more extreme hot days an individual encountered from Days 8 to 15 post‐hatch, the more likely they were to fledge at least one chick (Figure 5b).

#### 3.2.3 Proportion fledged

**FIGURE 6:**
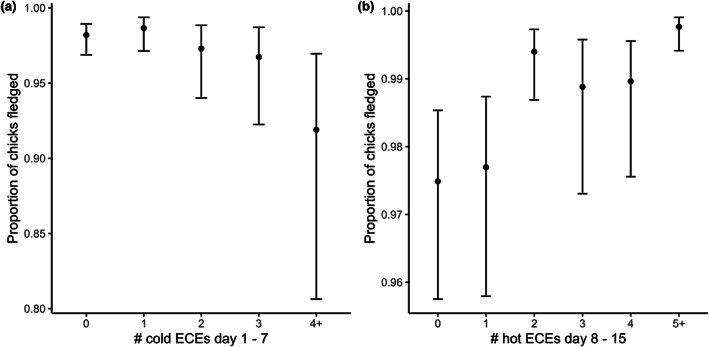
The proportion of great tit chicks that successfully survived to fledging declined as the number of cold extreme climatic events (ECEs) in the first week post‐hatching increased. In contrast, experiencing multiple hot ECEs during the period of peak chick food requirements (Days 8–15 post‐hatch) was associated with higher fledging success (*N* = 11,264 breeding attempts). Shown are model predictions and associated credible intervals.

We found evidence for effects of both cold and hot ECEs on the proportion of chicks that pairs successfully fledged, though these effects were only seen in specific phases of reproduction. For example, when we considered the number of cold ECEs encountered by birds between Days 1 and 7 post‐hatch, fledging success declined from 98% (95% CI = 96.9–98.9) for individuals that did not experience an ECE to 91.9% (95% CI = 80.6–97.0) for those that experienced four or more ECEs (Figure 6a). When we considered the number of hot ECEs between Days 8 and 15 post‐hatch, fledging success increased by 2.3% for birds that experienced five or more ECEs compared to birds that did not experience a high temperature ECE (Figure 6b).

### 3.3 Interactions between ECEs

**FIGURE 7:**
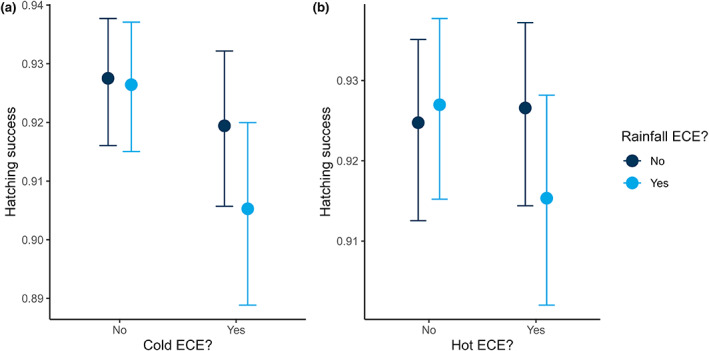
Encountering both a cold/hot extreme climatic event (ECE) and a high rainfall ECE during incubation had a larger negative impact on hatching success than encountering either a temperature ECE or a high rainfall ECE in isolation (*N* = 11,339 breeding attempts). Shown are model predictions and associated credible intervals.

**FIGURE 8:**
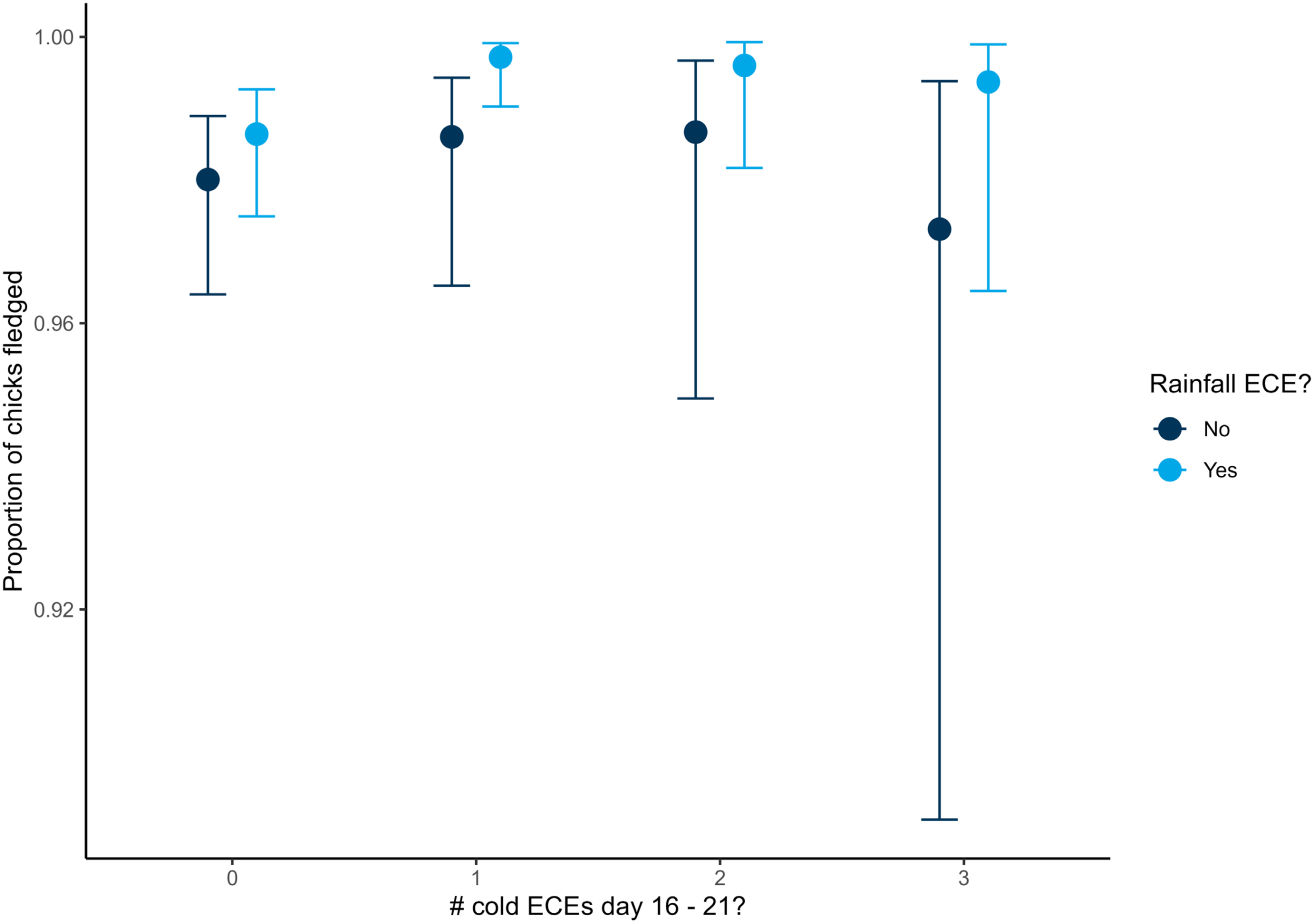
Encountering high rainfall extreme climatic events (ECEs) was beneficial to fledging success when combined with a cold ECE between days 16 and 21 (*N* = 11,264 breeding attempts). Shown are model predictions and associated credible intervals.

We found evidence for some interactions between temperature and rainfall ECEs, with interactions depending on the period considered. Focusing on the period between the start of incubation and hatching, we found that the combination of a high rainfall ECE and a cold/hot ECE was associated with reduced hatching success (Figure 7, Table S5). In contrast, high rainfall ECEs generally had a positive effect on reproductive success when combined with cold/hot ECEs post‐hatching (Table S6). For example, the probability of fledging at least one chick and the proportion of a brood that fledged were higher for individuals that encountered both a high rainfall ECE and a cold ECE between Days 16 and 21 than for individuals that only experienced a cold ECE (Table S6). Although we also found that birds encountering higher frequencies of cold ECEs were more likely to fledge more of their chicks if they also encountered a high rainfall ECE between Days 16 and 21 (Figure 8), this result should be treated with caution as for some ECE frequencies credible intervals did overlap zero (Table S7).

## 4 DISCUSSION

Climate change is already leading to increases in the frequency and severity of ECEs worldwide, yet our understanding of how such changes impact animal populations is limited. Much of the work to date has focused on the effects of single extreme events (see figure 1b in Bailey & van de Pol, 2016), which although valuable, do not allow us to look at critical factors that may shape the consequences of ECEs, such as the effects of ECE timing, cumulative effects of multiple ECEs, and interactions between different ECE types. However, some studies have begun to shed light on how the timing or duration of ECEs might mediate effects (e.g. Descamps et al., 2015; Marrot et al. 2017), and how the responses to ECEs may depend on other climatic factors (e.g. Bourne et al., 2020; Pattinson et al., 2022). Such information will be critical to predict the impacts of climate change into the future and understand how individuals and populations may respond. Using long‐term breeding data in combination with longitudinal climate data, we show that the reproductive success of great tits in a six decade population study is affected by high temperature ECEs, low temperature ECEs, and high rainfall ECEs, but is most negatively impacted by extreme low temperatures. We also show that effects of ECEs varied depending on when they occurred during breeding, according to their frequency, and were modified by the occurrence of other types of ECEs. Thus, our work demonstrates that to fully understand the effects of ECEs on natural populations, we will need to consider when they occur, how often they occur, and whether they occur alongside other ECEs.

As may be expected due to increasing average temperature at the global and local scales (Charmantier et al., 2008; Cole et al., 2021), we found that in this population the frequency of extreme hot days during spring (April–June) has increased over time, whilst the frequency of extreme cold days has decreased. However, we also show that patterns in the likelihood of exposure of birds to these events show complex, and slightly counter‐intuitive patterns, particularly in the case of extreme cold days. We found that the probability of a breeding pair encountering extreme cold days early in breeding has actually increased over time due to temporal changes in laying date that have previously been documented in this population (Charmantier et al., 2008; Cole et al., 2021), echoing a similar result found by Shipley et al. (2020) in a study of tree swallows (*Tachycineta bicolor*). Although the Wytham population has so far adequately tracked changing phenology of their food supply via phenotypic plasticity (Charmantier et al., 2008), our work suggests that these plastic changes in response to changing average climate conditions have also led to changes in the exposure of birds to low temperature ECEs, which occur with higher frequency earlier in the calendar year, that we have shown to negatively impact reproductive success. This highlights the likely complex interplay between the effects of changes in average climate and changes in variability/extreme events and indicates the need to understand whether existing plastic responses will be adequate in the face of other climate changes, such as in the patterns of ECEs.

Phenotypic plasticity, because of its rapidity, is a key mechanism by which individuals can adjust to changing climatic conditions (Chevin et al., 2010). It is expected to provide a short‐term buffer to populations in the face of climate change, buying time for evolutionary changes that may facilitate adaptation over the longer term (Chevin et al., 2010; Ghalambor et al., 2007). There has been considerable work across a variety of taxa exploring the role of plasticity in enabling responses to changing average climate (e.g. Anderson et al., 2012; Charmantier et al., 2008; Réale et al., 2003; Sauve et al., 2019), but the potential for plasticity to enable responses to ECEs is less understood. Importantly, plasticity relies on changes being predictable, with individuals requiring cues that reliably predict future conditions to make an appropriate phenotypic adjustment (Tufto, 2000). Given the general assumption that ECEs are largely unpredictable, theory predicts that plasticity is unlikely to provide an effective means of response (Reed et al., 2010; Tufto, 2000). Indeed, we are beginning to gain empirical evidence that this is the case (Lescroël et al., 2014; Leung et al., 2020). Clearly, further work is needed to clarify if/when ECEs are unpredictable, as well as whether learning may facilitate responses even where events are unpredictable. However, given that great tits are short‐lived, with an average generation time of only 1.8 years in this population (Bouwhuis et al., 2009), learning may not provide a means of efficient adjustment in this population. Thus, it may be that continued plastic responses to average climate conditions in Wytham's great tits will lead to continued increases in the exposure of birds to low temperature ECEs.

The evidence for a temporal trend in the likelihood of birds encountering extreme cold days early in breeding is of particular interest given that we also found that extreme cold days were associated with marked reductions in reproductive success, especially when considering birds that had encountered multiple cold ECEs. This suggests that we may expect to see reductions in reproductive success over time, that unless offset by increased chick survival at later stages, such as post‐fledging, could lead to effects on population growth. However, predicting the population‐level consequences of such relationships is not straightforward given the many factors that feed into population growth and the potential for feedback mechanisms that offset observed negative effects on quantities such as reproductive success. For example, Reed et al. (2013) showed that reductions in fledgling production due to phenological mismatch can be offset by reductions in competition for fledglings upon leaving the nest, thereby leading to no influence of mismatch on population growth.

Given that the temporal trend in exposure to cold ECEs appears to be largely driven by the changes in laying date observed in this population (Charmantier et al., 2008; Cole et al., 2021), it raises the question of whether birds are differentially affected by cold ECEs depending on their laying date. Though birds can and do plastically adjust their laying date, it is also repeatable within individuals (e.g. Bourret & Garant, 2015; Thorley & Lord, 2015; Van Der Jeugd & McCleery, 2002). If, for example, early laying individuals are most likely to experience one or more low temperature ECEs, and thus have lower reproductive success, we may expect changes in ECE exposure to alter the selection occurring on laying date in this population. Few studies have explored the effects of ECEs on selection, but some examples demonstrate that ECEs can alter selection on traits such as body size (Brown & Brown, 1998), beak size (Grant & Grant, 1993), and laying date (Marrot et al., 2017). Clearly, the effects of ECEs on selection will depend on a variety of factors, including the selection acting via changing average conditions, the frequency with which ECEs occur, and the degree to which different ECE types have opposing effects on fitness. Note that we used a fixed definition of an ECE over a 56‐year period, which thus implicitly assumes (when testing fitness effects) that the effect of an ECE on individuals is constant over that time. Given a mean generation time of ~1.8 years (Bouwhuis et al., 2009), it is possible that some adaptation to ECEs could have occurred over this time. However, we consider this relatively unlikely, as fluctuating climatic conditions between years mean that any selection is not constant. Nevertheless, our work indicates the importance of considering the selective consequences of changes in average climate conditions and in variability/ECEs in tandem.

Our finding that extreme cold days had more pronounced negative effects on reproductive success than extreme hot days contrasts with results from a similar study on blue tits that found extreme hot days to be particularly detrimental to reproductive success (Marrot et al., 2017). This difference may be at least partly due to contrasting climatic conditions in the two study areas, and thus differences in the stresses imposed by high and low temperature ECEs as defined in the two studies. Although our definitions were similar (hot ECEs = daily anomaly ≥4.98°C in Marrot et al. 2017 and ≥4.51°C in our study; cold ECEs = daily anomaly ≤ −5.31°C in Marrot et al. 2017 and ≤ −4.45°C in our study), the severity of high temperature and low temperature ECEs is likely to differ between the populations given the higher average temperatures during spring (March–May) in Montpellier, Southern France (13.5°C) compared to Oxfordshire (9.1°C). For example, if we consider May, when many birds are raising chicks in our study population, a hot ECE as defined in our study equated to a day with a mean temperature of 16.1°C. Such temperatures are unlikely to be associated with behavioural and physiological changes that in turn impact fitness as such responses are rarely observed at temperatures below 30°C (Cunningham et al., 2013; du Plessis et al., 2012; Kruuk et al., 2015). In contrast, the lower average temperatures at Wytham likely mean that birds in our study faced colder extremes than those birds studied by Marrot et al. 2017, which may explain why we found marked consequences of cold ECEs whilst they did not. We are unsure of the precise mechanisms driving the detrimental effects of cold ECEs in our study; however, previous work has shown that cold temperatures impact caterpillar growth and development, and thus biomass (Perrins, 1991; van Noordwijk et al., 1995; Whitehouse et al., 2013). This, in combination with the potential for higher energy requirements of birds in cold conditions, may mean that adults and chicks were energetically constrained during a cold ECE. Similarly, a combination of cold and wet conditions can limit the insulation provided by feathers and thus impact survival (Gardner et al. 2017; Robinson et al., 2007). This may explain our finding that cold ECEs had a greater negative impact on hatching success when there was also a high rainfall ECE. Further work will be needed to pinpoint the precise physiological and behavioural mechanisms driving relationships between ECE occurrence or frequency and fitness outcomes, such as those we have found here.

Though our work served to highlight that different populations of the same species likely differ in ECE exposure and responses, it is also important to note that such differences are unlikely to remain static. Continued climate change is expected to lead to increases in average temperature and/or variance in temperature, with different regions likely to be affected to different degrees. Such changes mean that ECEs defined based on their deviation from long‐term averages, as in our analysis, will likely correspond to higher absolute temperature values over time, though the degree of change will likely vary between regions. Thus, we should expect changes in the frequency and severity of ECEs over time, with such changes likely to lead to differences in the observed impacts of ECEs on individuals and populations. This highlights the need for more studies examining the impacts of ECEs and the dependence of impacts on factors such as timing, frequency, and severity. However, it also underlines the importance of considering the ECE definition used. Indeed, there has already been considerable discussion around the importance of selecting an ECE definition and of clearly communicating the definition used (Bailey & van de Pol, 2016; Smith, 2011).

Though our work has shed light on potential interactions between ECE types, with the effects of both cold ECEs and hot ECEs on reproductive success differing when there was also at least one high rainfall ECE in the same period, our ability to study interactions between ECEs was limited due to the rarity of even single extreme events. Specifically, we were unable to test for interactions between the number of hot/cold ECEs and the number of high rainfall ECEs, or to determine whether interactions were dependent on the temporal proximity of different types of ECEs. Therefore, our results may not accurately represent the true extent of interactions between extreme temperature and rainfall events. This highlights the importance of collecting both long‐term climatic datasets and population data. Such datasets, in combination with experimental simulations of ECEs will likely be needed to uncover the potential effects of changing ECE patterns, and thus enable action before climatic changes are so severe that we observe extreme biological impacts.

In addition to developing our understanding of how ECEs may affect natural populations, there are other areas ripe for future research. In particular, there is a need for studies to explore the exposure to, and effects of, ECEs at the individual level. Although we characterized the exposure of individuals to ECEs by considering the timing of individual breeding attempts, we implicitly assumed that all birds experienced the same climatic conditions. Given that we expect local climate conditions to vary given a variety of factors including vegetation structure (Huryna & Pokorný, 2016), aspect (Bennie et al., 2008), and elevation (Vanwalleghem & Meentemeyer, 2009), it is sensible to expect variation in the exposure of individuals to ECEs given environmental heterogeneity. To our knowledge, there is little work investigating the potential for variation in microclimate or habitat quality to alter the exposure of individuals to ECEs or affect their ability to respond behaviourally or physiologically to ECEs. Such work will be important to understand whether spatial environmental heterogeneity will buffer against continued increases in ECE frequency and severity, as well as to understand the potential for individuals to use information gained through previous experience or via social relationships to adjust their space use, and thus exposure, accordingly. Indeed, we are just beginning to understand whether flexibility in movement behaviour may facilitate responses to ECEs (Abernathy et al., 2019; Bailey et al., 2017).

In summary, we show that the fitness consequences of ECEs are likely to depend on the timing of ECEs, their frequency, and interactions between different ECE types. We also show that plastic responses to changing average climate conditions may alter exposure to ECEs, thus emphasizing the importance of jointly considering responses to directional changes in climate and changes in climate variability. It is only in doing so that we can hope to fully understand the potential ecological consequences of anthropogenic climate change.

## CONFLICT OF INTEREST STATEMENT

The authors declare no conflicts of interest.

## Supporting information


Data S1.

## Data Availability

The data that support the findings of this study are openly available in Dryad at https://doi.org/10.5061/dryad.tht76hf3r.
