# Supplementary material for: Phenotypic plasticity increases exposure to extreme climatic events that reduce individual fitness
Source: Glob Chang Biol. 2023 Mar 19;29(11):2968–80. doi: 10.1111/gcb.16663 (PMC10947444; doi:10.1111/gcb.16663)
Supplement: Supplementary file 1 — Data S1. [file GCB-29-2968-s001.docx]

**SUPPORTING INFORMATION for Regan & Sheldon: Phenotypic plasticity increases exposure to extreme climatic events that reduce individual fitness**

**Table of contents**

Effects of using different time windows when defining ECEs ………………………………………….. Page 2

Grouping information for ECE frequency ………………………………………………………………………… Page 4

Outputs of models with main effects only ………………………………………………………………………. Page 5

Hatching success …………………………………………………………………………………………………. Page 5

Brood failure ……………………………………………………………………………………………………….. Page 5

Proportion fledged ………………………………………………………………………………………………. Page 6

Outputs of models including interaction terms ……………………………………………………………….. Page 7

Hatching success …………………………………………………………………………………………………. Page 7

Brood failure ……………………………………………………………………………………………………….. Page 7

Proportion fledged ………………………………………………………………………………………………. Page 9

**Effect of using different time windows when quantifying ECEs**

To check that our ECE definitions were robust to the time window used to calculate long term climatic averages, we compared ECEs calculated using the calendar month (as reported in the manuscript), a 30-day time window centred around the focal day, a 10-day time window centred around the focal day, and a 7-day time window centred around the focal day. Cut-offs for defining ECEs were similar regardless of the time window used (Table S1) and results of analyses were also consistent regardless of the time window used when quantifying ECEs. For example, the estimated effect of the presence/absence of a hot ECE on fledging success was comparable across the different definitions (Figure S1). Similarly, the estimated effects of multiple cold ECEs on fledging success was also comparable across the definitions (Figure S2).

**Figure S1.** Using time windows of different lengths when defining hot ECEs did little to affect estimates of the effect of hot ECEs on fledging success in each of the post-hatch periods. Shown are effect size estimates and 95% credible intervals from mixed models.

**Figure S2**. Using time windows of different lengths when defining cold ECEs had little effect on estimates of the effect of cold ECE frequency on fledging success in each of the post-hatch periods considered. Shown are effect sizes and 95% credible intervals from mixed models.

**Grouping information for ECE frequency analyses**

**Table S1**. Due to the rarity with which individuals experienced high frequencies of ECEs, we opted to group higher frequencies into a single factor level and consequently treat ECE frequency as a categorical variable in analyses. Green shaded areas correspond to values that were grouped into a single factor level for each type of ECE in each reproductive period.

| ECE type | Frequency | | | | | | | | | |
| --- | --- | --- | --- | --- | --- | --- | --- | --- | --- | --- |
|  | 0 | 1 | 2 | 3 | 4 | 5 | 6 | 7 | 8 | 9 |
| Incubation |  |  |  |  |  |  |  |  |  |  |
| Hot | 6917 | 1731 | 1293 | 730 | 220 | 95 | 315 | 33 | 4 | 1 |
| Cold | 8742 | 913 | 891 | 257 | 199 | 87 | 197 | 33 | 14 | 6 |
| Rainfall | 6290 | 3516 | 1104 | 185 | 130 | 113 | 1 | 0 | 0 | 0 |
| Day 1-7 |  |  |  |  |  |  |  |  |  |  |
| Hot | 9264 | 1008 | 424 | 271 | 154 | 94 | 103 | 21 | 0 | 0 |
| Cold | 9564 | 606 | 562 | 321 | 286 | 0 | 0 | 0 | 0 | 0 |
| Rainfall | 6778 | 3273 | 899 | 380 | 9 | 0 | 0 | 0 | 0 | 0 |
| Day 8-15 |  |  |  |  |  |  |  |  |  |  |
| Hot | 7879 | 1770 | 582 | 329 | 357 | 283 | 121 | 18 | 0 | 0 |
| Cold | 10447 | 461 | 207 | 103 | 93 | 12 | 16 | 0 | 0 | 0 |
| Rainfall | 6916 | 3258 | 751 | 414 | 0 | 0 | 0 | 0 | 0 | 0 |
| Day 16-21 |  |  |  |  |  |  |  |  |  |  |
| Hot | 9102 | 1251 | 471 | 236 | 172 | 94 | 13 | 0 | 0 | 0 |
| Cold | 10714 | 405 | 101 | 41 | 40 | 27 | 11 | 0 | 0 | 0 |
| Rainfall | 8538 | 2179 | 529 | 93 | 0 | 0 | 0 | 0 | 0 | 0 |

**Hatching success models**

**Table S2.** Posterior means and 95% credible intervals from mixed models estimating the relationship between ECE presence/absence and number, and the proportion of a clutch that hatched

|  | Hot ECE | | Cold ECE | | High rainfall ECE | |
| --- | --- | --- | --- | --- | --- | --- |
| Model term | Est | 95% CI | Est | 95% CI | Est | 95% CI |
| Standardised laying date | -0.08 | -0.11 – -0.05 | -0.08 | -0.10 – -0.05 | -0.08 | -0.10 – -0.05 |
| ECE yes/no | -0.06 | -0.12 – 0.01 | -0.19 | -0.27 – -0.10 | -0.05 | -0.11 – 0.01 |
| Standardised laying date | -0.08 | -0.10 – -0.05 | -0.08 | -0.10 – -0.05 | -0.08 | -0.10 – -0.05 |
| ECE frequency (1) | -0.08 | -0.16 – 0.01 | -0.04 | -0.15 – 0.07 | -0.03 | -0.09 – 0.05 |
| ECE frequency (2) | -0.04 | -0.13 – 0.06 | -0.27 | -0.38 – -0.16 | -0.12 | -0.23 – -0.02 |
| ECE frequency (3) | -0.01 | -0.13 – 0.11 | -0.31 | -0.48 – -0.14 | -0.02 | -0.20 – 0.19 |
| ECE frequency (4) | -0.20 | -0.39 – -0.01 | -0.37 | -0.56 – -0.19 | -0.20 | -0.43 – 0.03 |
| ECE frequency (5) | 0.03 | -0.13 – 0.18 | -0.44 | -0.65 – -0.23 | -0.30 | -0.55 – -0.06 |

**Brood failure models**

**Table S3A.** Posterior means and 95% credible intervals from mixed models estimating the relationship between ECE presence/absence and the probability that at least one chick successfully fledged

|  | Hot ECE | | Cold ECE | | High rainfall ECE | |
| --- | --- | --- | --- | --- | --- | --- |
| Model term | Est | 95% CI | Est | 95% CI | Est | 95% CI |
| Standardised laying date | -0.16 | -0.22 – -0.11 | -0.17 | -0.22 – -0.11 | -0.16 | -0.22 – -0.11 |
| ECE yes/no  (day 1-7) | 0.07 | -0.10 – 0.25 | -0.02 | -0.22 – 0.18 | 0.03 | -0.11 – 0.17 |
| ECE yes/no  (day 8 – 15) | 0.34 | 0.18 – 0.51 | -0.28 | -0.53 – -0.03 | -0.07 | -0.21 – 0.08 |
| ECE yes/no  (day 16 – 21) | -0.03 | -0.20 – 0.14 | 0.56 | 0.25 – 0.89 | 0.15 | -0.00 – 0.31 |

**Table S3B.** Posterior means and 95% credible intervals from mixed models estimating the relationship between ECE frequency and the probability that at least one chick successfully fledged

|  | Hot ECE | | Cold ECE | | High rainfall ECE | |
| --- | --- | --- | --- | --- | --- | --- |
| Model term | Est | 95% CI | Est | 95% CI | Est | 95% CI |
| Standardised laying date | -0.16 | -0.21 – -0.10 | -0.18 | -0.24 – -0.12 | -0.16 | -0.22 – -0.11 |
| Day 1-7 |  |  |  |  |  |  |
| ECE frequency (1) | 0.01 | -0.20 – 0.22 | 0.29 | 0.01 – 0.57 | 0.06 | -0.09 – 0.22 |
| ECE frequency (2) | 0.12 | -0.17 – 0.42 | -0.01 | -0.32 – 0.29 | -0.08 | -0.33 – 0.18 |
| ECE frequency (3) | 0.42 | -0.01 – 0.87 | -0.30 | -0.68 – 0.09 | -0.40 | -0.78 – -0.02 |
| ECE frequency (4) | -0.05 | -0.56 – 0.47 | -0.78 | -1.18 – -0.36 |  |  |
| ECE frequency (5) | 0.45 | 0.00 – 0.89 |  |  |  |  |
| Day 8-15 |  |  |  |  |  |  |
| ECE frequency (1) | 0.15 | -0.04 – 0.34 | -0.30 | -0.62 – 0.01 | -0.13 | -0.28 – 0.02 |
| ECE frequency (2) | 0.63 | 0.33 – 0.93 | -0.27 | -0.73 – 0.20 | 0.19 | -0.09 – 0.46 |
| ECE frequency (3) | 0.24 | -0.12 – 0.62 | -0.42 | -1.06 – 0.28 | 0.10 | -0.32 – 0.51 |
| ECE frequency (4) | 0.52 | 0.16 – 0.90 | 0.09 | -0.57 – 0.80 |  |  |
| ECE frequency (5) | 0.94 | 0.55 – 1.32 |  |  |  |  |
| Day 16-21 |  |  |  |  |  |  |
| ECE frequency (1) | 0.04 | -0.17 – 0.26 | 0.63 | 0.29 – 0.99 | 0.20 | 0.02 – 0.36 |
| ECE frequency (2) | -0.09 | -0.37 – 0.19 | 0.52 | -0.24 – 1.37 | 0.06 | -0.24 – 0.36 |
| ECE frequency (3) | -0.02 | -0.40 – 0.38 | 0.28 | -0.38 – 1.01 | 0.04 | -0.61 – 0.70 |
| ECE frequency (4) | 0.46 | 0.03 – 0.91 |  |  |  |  |
| ECE frequency (5) | -0.07 | -0.54 – 0.39 |  |  |  |  |

**Proportion fledged models**

**Table S4A**. Posterior means and 95% credible intervals from mixed models estimating the relationship between ECE presence/absence and the proportion of a brood that fledged

|  | Hot ECE | | Cold ECE | | High rainfall ECE | |
| --- | --- | --- | --- | --- | --- | --- |
| Model term | Est | 95% CI | Est | 95% CI | Est | 95% CI |
| Standardised laying date | -0.48 | -0.60 – -0.35 | -0.48 | -0.60 – -0.35 | -0.45 | -0.58 – -0.33 |
| ECE yes/no (day 1-7) | 0.43 | 0.04 – 0.81 | -0.37 | -0.82 – 0.09 | -0.10 | -0.42 – 0.22 |
| ECE yes/no (day 8-15) | 0.78 | 0.42 – 1.16 | -0.58 | -1.11 – -0.07 | -0.17 | -0.49 – 0.15 |
| ECE yes/no (day 16-21) | -0.02 | -0.39 – 0.36 | 0.68 | 0.06 – 1.30 | 0.54 | 0.21 – 0.89 |

**Table S4B.** Posterior means and 95% credible intervals from mixed models estimating the relationship between ECE frequency and the proportion of a brood that fledged

|  | Hot ECE | | Cold ECE | | High rainfall ECE | |
| --- | --- | --- | --- | --- | --- | --- |
| Model term | Est | 95% CI | Est | 95% CI | Est | 95% CI |
| Standardised laying date | -0.47 | -0.60 – -0.34 | -0.49 | -0.61 – -0.37 | -0.45 | -0.58 – -0.32 |
| Day 1-7 |  |  |  |  |  |  |
| ECE frequency (1) | 0.16 | -0.34 – 0.63 | 0.30 | -0.28 – 0.88 | -0.03 | -0.35 – 0.32 |
| ECE frequency (2) | 0.02 | -0.63 – 0.69 | -0.40 | -1.07 – 0.28 | -0.11 | -0.68 – 0.47 |
| ECE frequency (3) | 1.12 | 0.27 – 1.99 | -0.60 | -1.37 – 0.21 | -1.08 | -1.85 – -0.29 |
| ECE frequency (4) | 0.67 | -0.31 – 1.67 | -1.56 | -2.43 – -0.69 |  |  |
| ECE frequency (5) | 1.21 | 0.22 – 2.18 |  |  |  |  |
| Day 8-15 |  |  |  |  |  |  |
| ECE frequency (1) | 0.08 | -0.36 – 0.54 | -0.55 | -1.20 – 0.11 | -0.37 | -0.70 –-0.03 |
| ECE frequency (2) | 1.44 | 0.77 – 2.07 | -0.71 | -1.55 – 0.17 | 0.38 | -0.19 – 0.98 |
| ECE frequency (3) | 0.83 | 0.09 – 1.62 | -0.45 | -1.58 – 0.64 | 0.61 | -0.18 – 1.42 |
| ECE frequency (4) | 0.89 | 0.14 – 1.63 | 0.03 | -1.11 – 1.23 |  |  |
| ECE frequency (5) | 2.39 | 1.61 – 3.18 |  |  |  |  |
| Day 16-21 |  |  |  |  |  |  |
| ECE frequency (1) | 0.13 | -0.32 – 0.57 | 0.73 | 0.07 – 1.41 | 0.51 | 0.16 – 0.87 |
| ECE frequency (2) | -0.21 | -0.85 – 0.42 | 0.68 | -0.44 – 1.81 | 0.54 | -0.13 – 1.17 |
| ECE frequency (3) | 0.33 | -0.53 – 1.17 | 0.05 | -1.20 – 1.32 | 0.83 | -0.37 – 2.00 |
| ECE frequency (4) | 0.25 | -0.66 – 1.13 |  |  |  |  |
| ECE frequency (5) | -0.95 | -1.99 – 0.18 |  |  |  |  |

**Interactions**

**Hatching success**

**Table S5.** Posterior means and 95% credible intervals from mixed models estimating the effect of interactions between temperature and rainfall ECEs on the proportion of a clutch that hatched

|  | Hot ECE | | Cold ECE | |
| --- | --- | --- | --- | --- |
| Model term | Est | 95% CI | Est | 95% CI |
| Standardised laying date | -0.08 | -0.11 – -0.06 | -0.08 | -0.10 – -0.05 |
| Rainfall ECE yes/no | 0.03 | -0.05 – 0.11 | -0.01 | -0.09 – 0.06 |
| Temp ECE yes/no | 0.03 | -0.06 – 0.11 | -0.11 | -0.22 – -0.01 |
| Interaction | -0.18 | -0.30 – -0.07 | -0.16 | -0.29 – -0.03 |
| Standardised laying date | -0.08 | -0.11 – -0.06 | -0.08 | -0.10 – -0.05 |
| Rainfall ECE yes/no | 0.03 | -0.05 – 0.12 | -0.01 | -0.08 – 0.06 |
| ECE frequency (1) | -0.02 | -0.13 – 0.10 | 0.07 | -0.07 – 0.22 |
| ECE frequency (2) | 0.14 | 0.01 – 0.26 | -0.16 | -0.34 – 0.01 |
| ECE frequency (3) | 0.04 | -0.11 – 0.18 | -0.38 | -0.59 – -0.16 |
| ECE frequency (4) | -0.18 | -0.38 – 0.01 | -0.23 | -0.47 – 0.00 |
| ECE frequency (5) | 0.04 | -0.12 – 0.21 | -0.45 | -0.68 – -0.21 |
| Int (Temp freq 1) | -0.13 | -0.29 – 0.03 | -0.27 | -0.46 – -0.07 |
| Int (Temp freq 2) | -0.36 | -0.53 – -0.19 | -0.15 | -0.36 – 0.06 |
| Int (Temp freq 3) | -0.12 | -0.34 – 0.10 | 0.19 | -0.14 – 0.51 |
| Int (Temp freq 4) | 0.09 | -0.55 – 0.80 | -0.32 | -0.66 – 0.01 |
| Int (Temp freq 5) | -0.07 | -0.33 – 0.19 | -0.01 | -0.40 – 0.39 |

**Brood failure**

**Table S6A**. Posterior means and 95% credible intervals from mixed models estimating the effect of interactions between the presence/absence of temperature and rainfall ECEs on the probability of at least one chick successfully fledging

|  | Hot ECE | | Cold ECE | | |
| --- | --- | --- | --- | --- | --- |
| Model term | Est | 95% CI | | Est | 95% CI |
| Standardised laying date | -0.17 | -0.22 – -0.11 | | -0.17 | -0.23 – -0.11 |
| Rain ECE yes/no (day 1-7) | 0.11 | -0.05 – 0.26 | | 0.05 | -0.11 – 0.21 |
| Temp ECE yes/no (day 1-7) | 0.15 | -0.04 – 0.45 | | -0.02 | -0.25 – 0.21 |
| Int (day 1-7) | -0.30 | -0.64 – 0.05 | | -0.00 | -0.38 – 0.38 |
| Rain ECE yes/no (day 1-7) | -0.11 | -0.28 – 0.05 | | -0.05 | -0.20 – 0.10 |
| Temp ECE yes/no (day 8-15) | 0.22 | 0.03 – 0.42 | | -0.18 | -0.46 – 0.11 |
| Int (day 8 – 15) | 0.32 | 0.01 – 0.63 | | 0.02 | -0.45 – 0.51 |
| Rain ECE yes/no (day 16-21) | 0.11 | -0.07 – 0.29 | | 0.05 | -0.11 – 0.22 |
| Temp ECE yes/no (day 16-21) | -0.01 | -0.19 – 0.17 | | 0.24 | -0.12 – 0.60 |
| Int (day 16-21) | -0.07 | -0.42 – 0.28 | | 0.99 | 0.40 – 1.64 |

**Table S6B.** Posterior means and 95% credible intervals from mixed models estimating the effect of interactions between the frequency of temperature ECEs and the presence/absence of rainfall ECEs on the probability of at least one chick successfully fledging

|  | Hot ECE | | Cold ECE | |
| --- | --- | --- | --- | --- |
| Model term | Est | 95% CI | Est | 95% CI |
| Standardised laying date | -0.17 | -0.22 – -0.11 | -0.18 | -0.24 – -0.12 |
| Day 1-7 |  |  |  |  |
| ECE frequency (1) | 0.15 | -0.12 – 0.42 | 0.33 | 0.00 – 0.66 |
| ECE frequency (2) | 0.17 | -0.17 – 0.51 | 0.04 | -0.36 – 0.47 |
| ECE frequency (3) | 0.35 | -0.12 – 0.83 | -0.24 | -0.63 – 0.17 |
| ECE frequency (4) | 0.00 | -0.51 – 0.54 | -0.83 | -1.25 – -0.43 |
| ECE frequency (5) | 0.41 | -0.05 – 0.89 |  |  |
| Int (Temp freq 1) | -0.34 | -0.74 - 0.08 | -0.26 | -0.82 – 0.32 |
| Int (Temp freq 2) | -0.32 | -0.94 – 0.33 | -0.11 | -0.66 – 0.44 |
| Int (Temp freq 3) | 0.82 | -0.45 – 2.19 | -0.39 | -1.56 – 0.83 |
| Int (Temp freq 4) | -0.87 | -2.05 – 0.36 | 1.08 | 0.08 – 2.19 |
| Int (Temp freq 5) | 0.35 | -0.97 – 1.82 |  |  |
| Day 8-15 |  |  |  |  |
| ECE frequency (1) | -0.02 | -0.24 – 0.22 | -0.18 | -0.52 – 0.17 |
| ECE frequency (2) | 0.57 | 0.26 – 0.89 | -0.02 | -0.64 – 0.64 |
| ECE frequency (3) | 0.18 | -0.22 – 0.60 | -0.31 | -1.01 – 0.41 |
| ECE frequency (4) | 0.38 | -0.00 – 0.78 | 0.01 | -0.75 – 0.82 |
| ECE frequency (5) | 0.89 | 0.49 – 1.30 |  |  |
| Int (Temp freq 1) | 0.40 | 0.03 – 0.77 | -0.15 | -0.76 – 0.47 |
| Int (Temp freq 2) | 0.15 | -0.45 – 0.81 | -0.28 | -1.15 – 0.59 |
| Int (Temp freq 3) | 0.60 | -0.30 – 1.59 | 0.22 | -1.11 – 1.64 |
| Int (Temp freq 4) | 0.79 | -0.25 – 1.95 | 0.68 | -0.53 – 1.93 |
| Int (Temp freq 5) | 0.52 | -0.61 – 1.74 |  |  |
| Day 16-21 |  |  |  |  |
| ECE frequency (1) | 0.07 | -0.17 – 0.31 | 0.33 | -0.06 – 0.73 |
| ECE frequency (2) | -0.04 | -0.35 – 0.26 | 0.27 | -0.60 – 1.20 |
| ECE frequency (3) | 0.05 | -0.36 – 0.48 | 0.11 | -0.74 – 1.00 |
| ECE frequency (4) | 0.51 | 0.01 – 1.02 |  |  |
| ECE frequency (5) | 0.00 | -0.50 – 0.49 |  |  |
| Int (Temp freq 1) | -0.19 | -0.58 – 0.22 | 0.99 | 0.27 – 1.74 |
| Int (Temp freq 2) | -0.51 | -1.49 – 0.44 | 0.62 | -0.67 – 1.99 |
| Int (Temp freq 3) | 0.12 | -1.00 – 1.30 | 0.55 | -0.49 – 1.59 |
| Int (Temp freq 4) | -0.19 | -1.20 – 0.91 |  |  |
| Int (Temp freq 5) | -0.00 | -1.93 – 1.98 |  |  |

**Proportion fledged**

**Table S7A.** Posterior means and 95% credible intervals from mixed models estimating the effect of interactions between the presence/absence of temperature and rainfall ECEs on the proportion of a brood that successfully fledged

|  | Hot ECE | | Cold ECE | | |
| --- | --- | --- | --- | --- | --- |
| Model term | Est | 95% CI | | Est | 95% CI |
| Standardised laying date | -0.47 | -0.60 – -0.34 | | -0.48 | -0.61 – -0.36 |
| Rain ECE yes/no (day 1-7) | 0.04 | -0.31 – 0.34 | | 0.01 | -0.34 – 0.37 |
| Temp ECE yes/no (day 1-7) | 0.55 | 0.11 – 0.97 | | -0.21 | -0.71 – 0.29 |
| Int (day 1-7) | -0.60 | -1.37 – 0.15 | | -0.49 | -1.25 – 0.26 |
| Rain ECE yes/no (day 8-15) | -0.20 | -0.54 – 0.15 | | -0.16 | -0.49 – 0.18 |
| Temp ECE yes/no (day 8-15) | 0.61 | 0.17 – 1.04 | | -0.32 | -0.90 – 0.27 |
| Int (day 8-15) | 0.22 | -0.41 – 0.84 | | -0.06 | -1.01 – 0.91 |
| Rain ECE yes/no (day 16-21) | 0.42 | 0.04 – 0.79 | | 0.36 | 0.00 – 0.71 |
| Temp ECE yes/no (day 16-21) | 0.00 | -0.41 – 0.41 | | 0.11 | -0.57 – 0.78 |
| Int (day 16-21) | -0.07 | -0.79 – 0.63 | | 1.73 | 0.67 – 2.78 |

**Table S7B.** Posterior means and 95% credible intervals from mixed models estimating the effect of interactions between the frequency of temperature ECEs and the presence/absence of rainfall ECEs on the proportion of a brood that successfully fledged

|  | Hot ECE | | Cold ECE | |
| --- | --- | --- | --- | --- |
| Model term | Est | 95% CI | Est | 95% CI |
| Standardised laying date | -0.47 | -0.60 – -0.34 | -0.49 | -0.62 – -0.36 |
| Day 1-7 |  |  |  |  |
| ECE frequency (1) | 0.33 | -0.22 – 0.89 | 0.38 | -0.24 – 1.01 |
| ECE frequency (2) | -0.01 | -0.69 – 0.66 | 0.10 | -0.71 – 0.91 |
| ECE frequency (3) | 1.09 | 0.21 – 1.97 | -0.42 | -1.21 – 0.38 |
| ECE frequency (4) | 0.58 | -0.41 – 1.66 | -1.55 | -2.47 – -0.65 |
| ECE frequency (5) | 1.09 | 0.13 – 2.09 |  |  |
| Int (Temp freq 1) | -0.49 | -1.32 - 0.42 | -0.84 | -1.93 – 0.22 |
| Int (Temp freq 2) | -0.35 | -1.59 – 0.92 | -1.03 | -2.08 – -0.03 |
| Int (Temp freq 3) | 0.10 | -1.50 – 1.70 | -0.38 | -2.10 – 1.33 |
| Int (Temp freq 4) | -0.99 | -2.74 – 0.71 | 1.42 | -0.16 – 2.94 |
| Int (Temp freq 5) | 0.21 | -1.58 – 1.96 |  |  |
| Day 8-15 |  |  |  |  |
| ECE frequency (1) | -0.14 | -0.66 – 0.38 | -0.37 | -1.06 – 0.32 |
| ECE frequency (2) | 1.31 | 0.65 – 2.02 | -0.49 | -1.52 – 0.51 |
| ECE frequency (3) | 0.58 | -0.26 – 1.38 | -0.23 | -1.38 – 0.93 |
| ECE frequency (4) | 0.58 | -0.20 – 1.38 | 0.23 | -0.96 – 1.45 |
| ECE frequency (5) | 2.23 | 1.42 – 3.06 |  |  |
| Int (Temp freq 1) | 0.30 | -0.41 – 1.01 | -0.39 | -1.60 – 0.85 |
| Int (Temp freq 2) | 0.35 | -0.84 – 1.54 | -0.19 | -1.52 – 1.14 |
| Int (Temp freq 3) | 0.69 | -0.64 – 2.09 | 0.32 | -1.47 – 2.12 |
| Int (Temp freq 4) | 1.07 | -0.31 – 2.47 | 0.07 | -1.48 – 1.62 |
| Int (Temp freq 5) | 0.38 | -1.01 – 1.81 |  |  |
| Day 16-21 |  |  |  |  |
| ECE frequency (1) | 0.22 | -0.29 – 0.71 | 0.36 | -0.42 – 1.07 |
| ECE frequency (2) | -0.22 | -0.89 – 0.45 | 0.43 | -0.83 – 1.69 |
| ECE frequency (3) | 0.37 | -0.50 – 1.26 | -0.30 | -1.60 – 1.08 |
| ECE frequency (4) | 0.36 | -0.59 – 1.28 |  |  |
| ECE frequency (5) | -0.86 | -1.90 – 0.24 |  |  |
| Int (Temp freq 1) | -0.50 | -1.29 – 0.32 | 1.21 | 0.02 – 2.41 |
| Int (Temp freq 2) | 0.13 | -1.31 – 1.58 | 0.82 | -0.73 – 2.29 |
| Int (Temp freq 3) | 0.27 | -1.29 – 1.90 | 1.07 | -0.41 – 2.57 |
| Int (Temp freq 4) | -0.34 | -1.83 – 1.14 |  |  |
| Int (Temp freq 5) | -0.00 | -2.03 – 2.02 |  |  |
